# Patient Characteristics, Management, and Predictors of Outcome from Severe Community-Onset Staphylococcal Sepsis in Northeast Thailand: A Prospective Multicenter Study

**DOI:** 10.4269/ajtmh.16-0606

**Published:** 2017-05-03

**Authors:** T. Eoin West, Chanthiwa Wikraiphat, Sarunporn Tandhavanant, Pitchayanant Ariyaprasert, Pornpan Suntornsut, Shawna Okamoto, Weera Mahavanakul, Pramot Srisamang, Sunchai Phiphitaporn, Jirasak Anukunananchai, Ploenchan Chetchotisakd, Sharon J. Peacock, Narisara Chantratita

**Affiliations:** 1 Division of Pulmonary and Critical Care Medicine, Department of Medicine, University of Washington, Seattle, Washington; 2 International Respiratory and Severe Illness Center, University of Washington, Seattle, Washington; 3 Department of Microbiology and Immunology, Faculty of Tropical Medicine, Mahidol University, Bangkok, Thailand; 4 Mahidol-Oxford Tropical Medicine Research Unit, Faculty of Tropical Medicine, Mahidol University, Bangkok, Thailand; 5 Department of Medicine, Sunpasitthiprasong Hospital, Ubon Ratchathani, Thailand; 6 Department of Pediatrics, Sunpasitthiprasong Hospital, Ubon Ratchathani, Thailand; 7 Department of Medicine, Udon Thani Regional Hospital, Udon Thani, Thailand; 8 Department of Medicine, Khon Kaen Regional Hospital, Khon Kaen, Thailand; 9 Department of Medicine, Faculty of Medicine, Khon Kaen University, Khon Kaen, Thailand; 10 London School of Hygiene and Tropical Medicine, London, United Kingdom

## Abstract

*Staphylococcus aureus* infection is a persistent threat in resource-restricted settings in southeast Asia but informative data about this disease remain limited. We analyzed characteristics, management, and predictors of outcome in severely septic patients with community-onset *S. aureus* infection in northeast Thailand. We performed a prospective, multicenter observational cohort study of community-onset *S. aureus* sepsis in four referral hospitals recruiting patients at least 14 years of age admitted between March 2010 and December 2013. One hundred and nineteen patients with severe staphylococcal sepsis were enrolled. Diabetes was the most common underlying condition. Methicillin-resistant infection was rare. Twenty-eight-day mortality was 20%. Ninety-two percent of patients received appropriate antibiotic therapy and 82% were administered intravenous fluids on the first hospital day, although only 14% were managed in an intensive care unit (ICU). On univariable analysis, clinical variables at enrollment significantly associated with death at 28 days were coagulopathy or respiratory failure. Plasma interleukin (IL)-8 concentration alone accurately predicted mortality (area under the receiver operating curve = 0.82, 95% confidence interval = 0.73–0.90). In multivariable analysis, addition of IL-8 concentration to a mortality prediction model containing clinical variables further improved the predictive ability of the model. We conclude that severe staphylococcal sepsis in northeast Thailand causes significant mortality. Diabetes is a common preexisting condition and most patients are managed outside the ICU even if they receive vasoactive/inotropic agents or mechanical ventilation. While clinical factors apparent on presentation including coagulopathy and respiratory failure predict death, plasma IL-8 improves this prediction.

## Introduction

*Staphylococcus aureus* is an established pathogen in resource-restricted regions of southeast Asia^1^–^4^ and an increasingly important contributor to the substantial global burden of severe sepsis—the syndrome of infection accompanied by a dysregulated host inflammatory response and organ dysfunction.^5^,^6^ *Staphylococcus aureus* expresses numerous virulence determinants that result in infection of the skin and soft tissues, respiratory tract, bones, joints, and endovasculature leading to sepsis.^7^,^8^ Yet few studies address management of hospitalized patients with severe sepsis—due to *S. aureus* or to other pathogens—in resource-restricted settings in southeast Asia or elsewhere. In addition to appropriate antibiotic therapy and drainage of infected spaces, intravenous fluids, vasopressor and inotropic agents, and mechanical ventilation are often required for these individuals. In a resource-restricted environment, robust data about presentation, management, and outcomes of these patients are essential to inform strategies to allocate scarce resources judiciously and improve outcomes.^9^,^10^ Central to this process is understanding what factors may predict outcome. Presently, the predictors of outcomes such as death from severe sepsis in resource-restricted settings are largely unknown.

Thailand is a middle-income country in southeast Asia (https://datahelpdesk.worldbank.org/knowledgebase/articles/906519). The poorest and most populous area is in the northeast (http://documents.worldbank.org/curated/en/2005/11/7283755/thailand-northeast-economic-development-report). Here, studies of community-acquired bacteremia show that *S. aureus* is the most common Gram-positive etiology and the third most common etiology overall after *Escherichia coli* and *Burkholderia pseudomallei*.^11^,^12^ In Cambodia, *S. aureus* is the most common Gram-positive etiology of bacteremia.^2^,^3^ Analysis of resistance patterns of community-acquired bacteremia isolates in northeast Thailand indicates that methicillin-resistant *S. aureus* (MRSA) is infrequent.^12^ This contrasts with data from Cambodia, where MRSA is more common.^2^,^3^ A single center study in northeast Thailand from 2006 to 2007 that evaluated outcome following invasive *S. aureus* infection identified a 26% mortality rate associated with this disease.^13^ Nineteen percent of these cases reflected hospital-acquired infection, and the mortality rate was significantly higher (52%) in this subset.^13^ To better characterize the impact of community-onset *S. aureus* sepsis at a regional level, we subsequently conducted a prospective multicenter study at four hospitals in three northeastern Thai provinces. These hospitals all have intensive care units (ICUs) where care is provided by specialized teams of doctors and nurses in higher provider-to-patient ratios. Patients at all hospitals can receive mechanical ventilation and vasoactive agents in the ICUs and on the general wards. Mechanical ventilators may be powered by gas (pneumatic) or electricity. Some data from this study have previously been reported, including our finding that some isolates identified as *S. aureus* are in fact *Staphylococcus argenteus*, a new pathogenic species that is misidentified as *S. aureus* in clinical diagnostic laboratories using standard identification tests.^14^ In light of the lack of distinction between these organisms in the clinical diagnostic laboratories, we included both *S. aureus* and *S. argenteus* isolates in this study.

We used this study cohort to pursue three objectives. First, we aimed to characterize patients presenting with severe community-onset staphylococcal sepsis in northeast Thailand, including their clinical management and outcome. Second, we sought to identify clinical predictors of death in this setting. Third, we evaluated whether biological markers could augment the clinical prediction of death. Our findings provide key insights into severely ill patients with staphylococcal infection in a resource-restricted setting in southeast Asia.

## Materials and Methods

### Study setting and design.

A prospective observational study of community-onset invasive staphylococcal sepsis was conducted at four hospitals across northeast Thailand between March 2010 and December 2013. Study sites were Sunpasitthiprasong Hospital, Ubon Ratchathani; Udon Thani Hospital, Udon Thani; Srinagarind Hospital, Khon Kaen; and Khon Kaen Hospital, Khon Kaen. Potential study patients were identified by daily screening at each hospital diagnostic microbiology laboratory for clinical samples taken from a normally sterile site that grew a pure culture identified by the routine microbiology laboratory as *S. aureus* using methods derived from the Work Manual for Laboratory Identification of Bacteria and Fungi for Regional and General Hospitals, Department of Medical Science, Ministry of Public Health, Thailand. Subsequently, 58/311 (19%) of available isolates tested were reidentified in a research laboratory as *S. argenteus* using pulsed-field gel electrophoresis and multilocus sequence typing.^14^ Inclusion criteria were as follows: age at least 14 years (age of admission to adult wards), community-onset infection (positive culture taken within 2 days of hospital admission, or after 2 days when sampling was delayed from a patient admitted with suspected infection), and at least two of four systemic inflammatory response syndrome criteria met within 48 hours of culture (the definition of sepsis at the time the study was performed).^15^ Severe sepsis was defined at enrollment as systolic blood pressure < 90 or use of vasoactive agents, creatinine ≥ 2 mg/dL without a history of preexisting renal disease, bilirubin ≥ 2 mg/dL without a history of preexisting liver disease, platelet count ≤ 100,000/μL, prothrombin time ≥ 20 seconds, respiratory failure requiring invasive mechanical ventilation, or Glasgow Coma Scale (GCS) ≤ 12.^16^ Laboratory data were available to make this determination as follows: creatinine, 312/327 patients; bilirubin, 134/327; platelet count, 323/327; and prothrombin time, 100/327. Exclusion criteria were other active coinfections potentially contributing to the clinical presentation, therapeutic immunosuppression with high-dose steroids or chemotherapy, and pregnancy. Patients were enrolled after providing written informed consent. A blood sample was drawn at the time of enrollment.

Clinical and microbiological information for the hospitalization was obtained from the medical records. Mortality was ascertained in person or by telephone 28 days from the day of admission. No participants were lost to follow-up.

### Biomarkers.

Tumor necrosis factor (TNF)-α, interleukin (IL)-1β, IL-6, and IL-8 concentrations in plasma obtained at enrollment were determined by enzyme-linked immunosorbent assay (TNF-α, IL-1β and IL-6, BD Bioscience, Franklin Lakes, NJ; IL-8, R and D Systems, Minneapolis, MN). Lower limits of detection were 7.8 pg/mL, 3.9 pg/mL, 4.7 pg/mL, and 15.6 pg/mL, respectively.

### Ethics.

Ethical approval was obtained from the following ethical and scientific review committees: Faculty of Tropical Medicine, Mahidol University (approval no. MUTM 2011-007-01); Sunpasitthiprasong Hospital, Ubon Ratchathani (approval no. 004/2553); Udon Thani Hospital, Udon Thani (approval no. 0027.102/2349); Khon Kaen Hospital, Khon Kaen; and Faculty of Medicine (Srinagarind Hospital), Khon Kaen University, Khon Kaen, Thailand (approval no. HE541113). Subjects or their representatives provided written informed consent.

### Statistical analysis.

Continuous variables are reported as the mean and standard deviation for normally distributed data or median and interquartile range (IQR) for nonnormally distributed data. Plasma cytokine concentrations were available for 118/119 patients with severe sepsis and were log_10_ transformed before analysis. Missing values for GCS and laboratory tests were treated as normal in the determination of organ failure as noted in Supplemental Table 1. Univariable analyses were performed with logistic regression. Multivariable analyses were performed with multiple logistic regression. Linearity of continuous variables was evaluated using the Box–Tidwell procedure, and IL-8 and IL-6 were log_10_ transformed. A model of clinical predictors of death was developed using a backward elimination procedure on all variables collected or derived at enrollment reported in the univariable analysis with *P* < 0.1. Variables were sequentially removed based on the highest *P* value, until all variables retained had *P* < 0.05. Sex was also included as a variable given that IL-8 concentrations were higher in men. Internal validation of the selected predictors in the candidate model was performed by conducting 100 bias corrected and accelerated bootstrap replications, and evaluating the confidence intervals (CIs) around the coefficients of the predictors. The model was evaluated for multicollinearity and specification error, and assessed using Hosmer and Lemeshow goodness of fit testing. Differences in the model including log_10_ IL-8 or log_10_ IL-6 were determined by examining the Akaike information criterion (AIC) and Bayesian information criterion (BIC), by performing a likelihood ratio test, and by comparing areas under the receiver operating curves (ROCs). All statistical analyses were conducted using Stata v14.2 (StataCorp LP, College Station, TX).

## Results

### Characteristics of enrolled patients.

**Figure 1.**
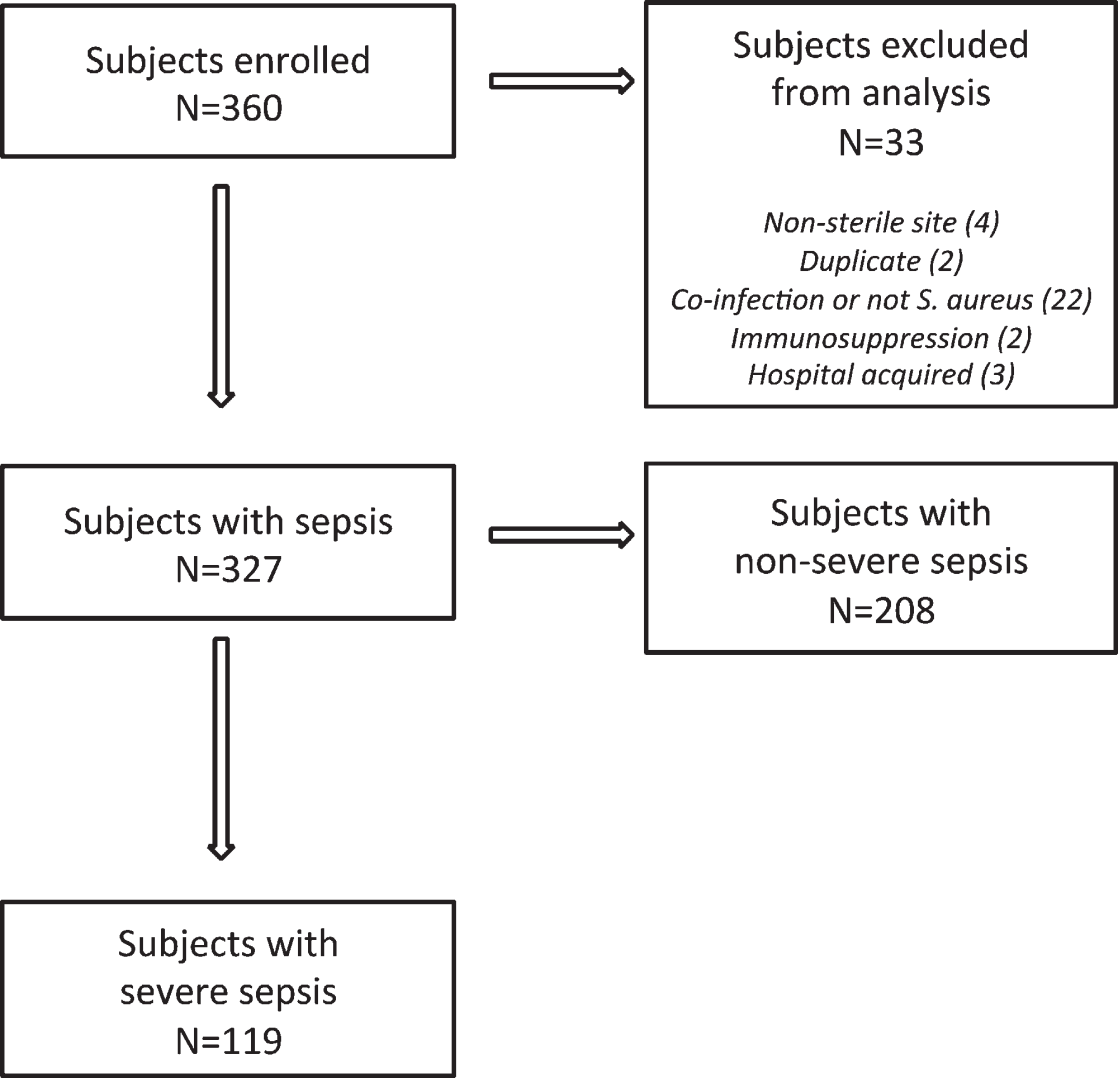
Study flow chart.

Three hundred and twenty-seven patients meeting *Staphylococcus* sepsis criteria were enrolled. Of these, 119 (36.4%) met the criteria for severe sepsis (Figure 1

). Clinical characteristics of the patients with severe sepsis are shown in Table 1. The median age was 58.5 years (IQR = 46–67) and 80 (67.2%) were male. The median duration of symptoms was 4 days (IQR = 2–7). Seventy-three (61.3%) patients had an underlying medical condition. The most common preexisting conditions were diabetes (*N* = 42, 35.3%) and kidney disease (*N* = 23, 19.3%). Seventy-six (63.9%) patients with severe sepsis had positive blood cultures at the time of enrollment; the remaining patients had positive cultures of pus specimens. Bacterial isolates from three patients (2.5%) were MRSA.

### Organ failure in patients with severe staphylococcal sepsis.

Organ failure in patients with severe sepsis at the time of enrollment is shown in Supplemental Table 1. Respiratory failure (*N* = 46, 38.7%) and shock (*N* = 45, 37.8%) were the most common manifestations of severe sepsis. Thrombocytopenia (*N* = 34, 28.6%) and acute kidney injury (*N* = 30, 25.2%) were also frequent.

### Clinical management and outcome of patients with severe staphylococcal sepsis.

Specific therapies provided for the management of severe sepsis, including antibiotics and drainage procedures, intravenous fluid resuscitation, and organ support measures are shown in Table 1. Most patients (*N* = 109, 91.9%) received antistaphylococcal antimicrobial therapy by the first day of hospital admission. Of the 10 patients in whom appropriate therapy was delayed or not initiated, two had MRSA. Vancomycin was administered to 21 (17.6%) patients. A drainage procedure was performed on 50 (42.0%) patients. Most patients (*N* = 97, 81.5%) received intravenous fluids on the first hospital day. Seventeen (14.3%) patients were admitted to ICUs; the remaining patients were managed on general wards. These patients did not differ by age, sex, or underlying medical condition. Only patients requiring mechanical ventilation were admitted to ICUs but the majority of patients (*N* = 34, 66.7%) requiring mechanical ventilation were cared for on the general wards.

Hospital mortality was 14.3% and 28-day mortality was 20.2% among severe sepsis patients (compared with 2.9% 28-day mortality in patients with nonsevere sepsis). One of three (33.3%) patients with MRSA died within the 28-day study period. Twenty-eight-day mortality was 52.9% in patients admitted to an ICU but 14.7% in patients managed on the general wards.

### Biomarkers in patients with severe staphylococcal sepsis.

**Figure 2.**
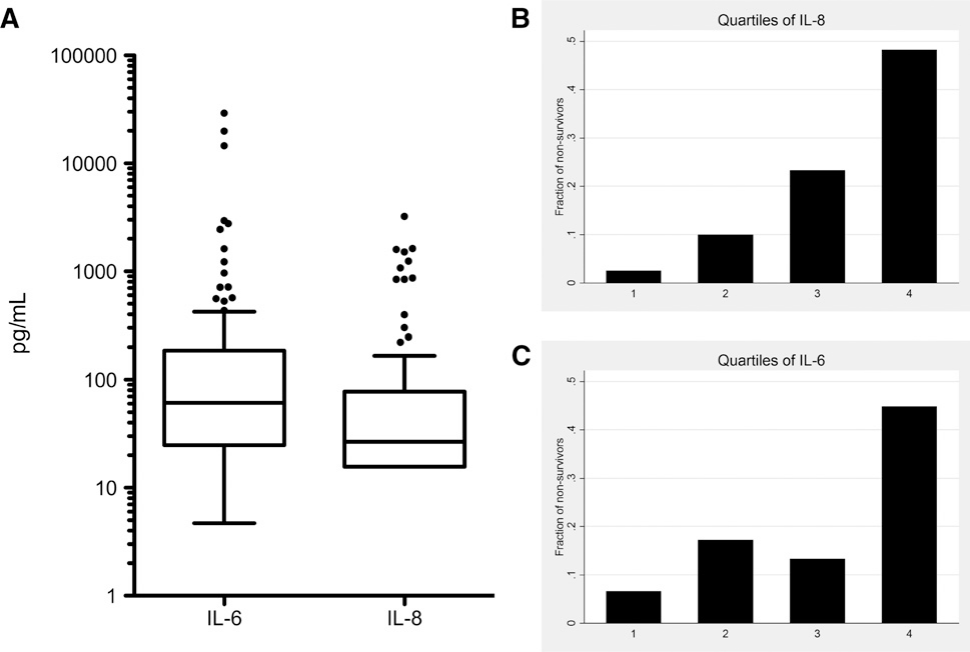
Plasma cytokine concentrations in patients with severe staphylococcal sepsis. (**A**) The distribution of interleukin (IL)-6 and IL-8 concentrations in study subjects. Boxes show 25th, 50th, and 75th percentiles. Bottom and top whiskers show 25th percentile minus 1.5 times the interquartile range (IQR) and 75th percentile plus 1.5 times the IQR, respectively. (**B**) and (**C**) Mortality in patients with severe staphylococcal sepsis by quartile of IL-8 (B) or IL-6 (C) concentrations. *P* for trend = 3.5 × 10^−6^ and *P* for trend = 0.001, respectively.

We measured the concentrations of four pro-inflammatory cytokines in patients' plasma drawn at the time of enrollment: TNF-α, IL-1β, IL-6, and IL-8. We chose these biomarkers because of previous reports of their utility for sepsis detection or prognostication.^17^ All but one TNF-α value and over 75% of IL-1β values were below the lower limit of detection of our assay so they were not analyzed further. In contrast, there was a marked range of IL-6 and IL-8 responses across several orders of magnitude (Figure 2A

).

### Univariable analysis of predictors of death in patients with severe staphylococcal sepsis.

We then assessed which clinical factors were associated with 28-day mortality among patients with severe staphylococcal sepsis (Table 2). In univariable analyses, no preexisting condition was significantly associated with mortality, although the odds ratio (OR) for death for patients with heart disease was 3.60 (95% CI = 0.89–14.62, *P* = 0.07). At the time of enrollment, the presence of coagulopathy (OR = 5.44, 95% CI = 1.78–16.59, *P* = 0.003), or respiratory failure (OR = 4.33, 95% CI = 1.67–11.23, *P* = 0.003) were significantly associated with death. The association of altered mental status with death did not reach statistical significance (OR = 2.97, 95% CI = 0.77–11.50, *P* = 0.12). During the hospitalization, any initiation of mechanical ventilation (OR = 7.48, 95% CI = 2.56–21.88, *P* < 0.01), or use of vasoactive agents (OR = 3.52, 95% CI = 1.38–8.98, *P* < 0.01) was associated with death, but receipt of a drainage procedure was protective (OR = 0.29, 95% CI = 0.11–0.85, *P* = 0.02). Patients admitted to an ICU had greatly increased odds of death (OR = 6.53, 95% CI = 2.17–19.58, *P* = 0.001).

**Figure 3.**
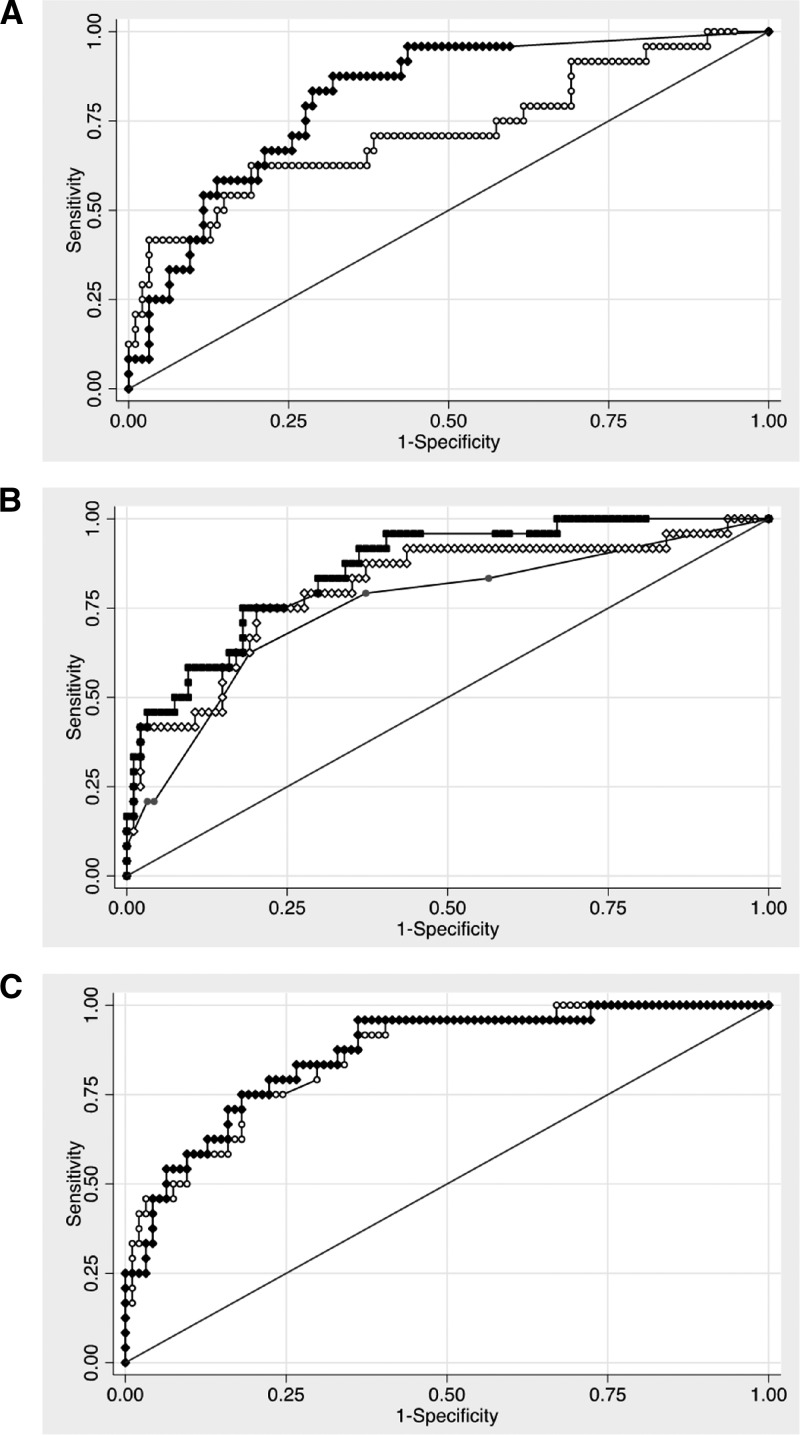
Receiver operating curves for prediction of death. (**A**) Log_10_ interleukin (IL)-6 (white circles) and log_10_ IL-8 (black diamonds). (**B**) A baseline model of clinical variables (grey circles) with the addition of either log_10_ IL-6 (white diamonds) or log_10_ IL-8 (black squares). (**C**) A model of clinical variables and log_10_ IL-8 (white circles) with the addition of log_10_ IL-6 (black diamonds).

We analyzed whether plasma cytokine concentrations predicted outcome in our cohort. Both IL-6 and IL-8 levels were strongly associated with death (OR = 3.91, 95% CI = 1.92–7.94, *P* = 1.7 × 10^−4^ and OR = 5.86, 95% CI = 2.52–13.63, *P* = 4.1 × 10^−5^), for log transformed IL-6 and IL-8concentrations, respectively. Quartiles of IL-8 showed a stepwise increase in mortality rate (P for trend = 3.5 × 10^−6^), whereas for IL-6, there was a more apparent threshold effect between the third and fourth quartiles (*P* for trend = 0.001) (Figure 2B and 2C). Next, we performed ROC analysis on the prediction of mortality by mediator concentrations. The AUROC for IL-6 was 0.72 (95% CI = 0.59–0.85) (Figure 3A

). The area under the ROC (AUROC) for IL-8 was 0.82 (95% CI = 0.73–0.90) (Figure 3A).

### Multivariable analysis of predictors of death in patients with severe staphylococcal sepsis.

We then performed multivariable regression of predictors of death among severe staphylococcal sepsis patients. To develop a model using clinical variables, we considered clinical data available at enrollment and included variables with *P* values < 0.1 in the initial model. We also included sex as IL-8 concentrations were higher in men. In the final model comprising clinical variables, coagulopathy and respiratory failure were independently associated with 28-day mortality (Table 3). The AUROC for mortality prediction by this baseline model was 0.75 (95% CI = 0.64–0.87) (Figure 3B).

To determine the additional contribution of IL-6 and IL-8 as predictive biomarkers in this model, we repeated the model including these mediators individually and together. We found that the model including IL-6 had lower AIC and BIC scores than the baseline model (Table 3) and the two models were significantly different by the likelihood ratio test (*P* = 0.004). The AUROC for mortality prediction by the model including IL-6 was 0.81 (95% CI = 0.70–0.91) (Figure 3B), which was not significantly different from the baseline model (*P* = 0.12). Including IL-8 in the model instead of IL-6 resulted in a greater reduction of AIC and BIC scores (Table 3), and this model was highly significantly different by the likelihood ratio test (*P* = 1.9 × 10^−5^). The AUROC for mortality prediction by the model including IL-8 was 0.86 (95% CI = 0.78–0.94) (Figure 3B), which was significantly different from the baseline model without IL-8 (*P* = 0.03). There was no added benefit of adding IL-6 to a model with clinical variables and IL-8; the likelihood ratio test *P* = 0.29 and AUROC was 0.86 (95%CI = 0.78–0.94), *P* = 0.60 (Figure 3C). These results indicate the added benefit of including IL-8, but not IL-6, together with clinical variables as predictors of mortality.

## Discussion

Gram-positive organisms are important etiologies of sepsis in high-income countries.^5^ The available evidence indicates that staphylococcal infection is also of increasing concern in resource-restricted settings in Asia, including northeast Thailand.^1^–^4^,^11^,^12^ To our knowledge, our prospective multicenter study is one of the first to provide important data characterizing severe staphylococcal sepsis in such a setting. The major findings from our study are that severe staphylococcal sepsis causes significant mortality despite appropriate antibiotic therapy; diabetes is a common preexisting condition; most patients are managed outside the ICU even if they receive vasoactive/inotropic agents and mechanical ventilation; and while clinical factors apparent on presentation including coagulopathy and respiratory failure predict death, plasma IL-8 significantly improves this prediction.

In this multicenter study the 28-day mortality rate from severe staphylococcal sepsis was 20%. A previous single center study in this region from 2006 to 2007 reported a mortality rate of 20% at 12 weeks in hospitalized patients with non-nosocomial invasive *S. aureus* infection (defined as *S. aureus* cultured from a sterile site).^13^ In a related analysis of patients with *S. aureus* bacteremia—including nosocomial infection—who met criteria for severe sepsis, the 28-day mortality rate was 53%.^18^ Together, these results underscore the significant burden of severe staphylococcal sepsis in northeast Thailand. They no doubt underestimate overall mortality, however, as these studies excluded patients who died before their cultures turned positive. The patients with severe staphylococcal sepsis in our study were older and mostly male. It is notable that nearly two thirds of patients had an underlying medical condition, and strikingly, diabetes was the predominant preexisting disease, present in over a third of patients. This not only reflects the increasing rates of noncommunicable chronic diseases such as diabetes worldwide,^19^ but may also indicate the impact of diabetes on susceptibility to bacterial infections such as *S. aureus*.^20^

The management of severely septic patients with staphylococcal infection requires clinician awareness to diagnose severe sepsis, promptly administer appropriate antibiotics, drain pus, fluid resuscitate, and rigorously support failing organs.^18^ Although adherence to these basic tenets of sepsis management is essential, recent studies of severe sepsis in high-resource settings indicate that there is little benefit to an invasive early goal-directed care protocol.^21^–^23^ Most of the patients in our study received appropriate antimicrobial therapy and intravenous fluids within the first hospital day as well as access to surgical care and other organ support measures where necessary. MRSA was an uncommon cause of infection in our study but vancomycin, available at all sites, was administered quite frequently. While ICUs are also available at all the study sites, the large majority of patients enrolled were managed outside an ICU. Patients admitted to an ICU were more likely to have respiratory failure and more likely to die. Severely septic patients are often managed in ICUs in high-resource settings. Our observations suggest that ICU admission in this study was restricted to the subset of sickest patients, but that the majority of patients managed on general wards nonetheless received necessary therapies.

We identified a number of risk factors for death from severe staphylococcal sepsis in northeast Thailand. In a multivariable analysis, the major clinical predictors on presentation to the hospital were coagulopathy and respiratory failure. These probably differ from a previous report of risk factors for death from *S. aureus* infection in northeast Thailand (in which age and cardiac disease were the presenting factors predicting death)^13^ due to our focus on community-onset infection and patients with severe sepsis. In addition to these clinical risk factors, we also sought a biomarker that might serve as a useful adjunct predictor. If sufficiently accurate, such a biomarker could be assayed in a point-of-care device and therefore used by clinicians at the bedside in resource-restricted settings. The chemokine IL-8 is a fundamental participant in many inflammatory processes. It is produced by many different cells and is well described as a biomarker predicting outcome in sepsis,^24^–^28^ but not all reports support its utility as a risk stratification tool.^29^ We are not aware that it has previously been evaluated extensively in staphylococcal sepsis. We found that plasma levels of IL-8 provide accurate prediction of death, both alone and—crucially—when combined with clinical variables. Indeed, inclusion of IL-8 in the regression model substantially augmented the prediction of death compared with clinical variables alone. While plasma IL-6, TNF-α, and IL-1β levels are also reported as predictors of death from sepsis,^30^ in our study the utility of IL-8 was much greater. Nonetheless, this finding requires external validation in other cohorts of severe staphylococcal sepsis. It also remains to be determined whether the characteristics of the clinical model will remain accurate in other causes of sepsis. If the predictive value of IL-8 is confirmed in these settings, it could serve to identify patients at high risk of death who deserve more intensive management upon recognition of sepsis. The low plasma concentrations of TNF-α and IL-1β in our cohort mirror the findings of several other investigators,^31^–^33^ but it is also possible that levels of these mediators may have been higher earlier in the clinical course.

Our study offers several strengths, including its prospective, multicenter design and excellent rate of follow-up. Our use of 28-day mortality rather than hospital mortality is especially relevant in this region where very ill patients are often taken home from the hospital to die. Our focus on staphylococcal sepsis addresses an important etiology of sepsis in the region, and it provides key insights into management of severe infection in provincial Thailand. Our investigation of IL-8 is pertinent as this mediator has not been thoroughly evaluated as a biomarker in staphylococcal sepsis.

The main limitation to our study was that we did not enroll patients until after a culture-proven diagnosis of staphylococcal infection. Therefore, enrollment did not occur for at least 48 hours into hospitalization and our figures are likely to underestimate early deaths. Some patients were enrolled even later when the bacterial isolation was further delayed. However, our mortality figures are similar to those in another prospective study of severe sepsis in this region that enrolled patients within 24 hours of admission (D Limmathurotsakul and TE West, unpublished data). Although we enrolled patients with infection identified by the clinical laboratory as *S. aureus,* we recently reported that a subset of these patients actually was infected with a related pathogen, *S. argenteus*.^14^ Clinical manifestations of *S. aureus* infection may be more severe than *S. argenteus* infection,^14^ but as these bacteria are not differentiated in routine practice in this setting, we did not consider this in our analysis. Finally, the timing of antibiotic and intravenous fluid administration in our study is reported in days, whereas other studies support the importance of these treatments within hours of sepsis onset.^34^

## Conclusions

We show that at least one in five patients with severe community-onset staphylococcal sepsis admitted to provincial hospitals in northeast Thailand country die. Many patients have diabetes, and most patients are managed outside ICUs. MRSA is uncommon. We define clinical risk factors for death and identify a highly predictive role for IL-8 that deserves further evaluation. These data expand our knowledge of severe staphylococcal sepsis in resource-restricted areas in southeast Asia.

## Supplementary Material

Supplemental Table.

## Figures and Tables

**Table 1:** Baseline characteristics and management of patients with severe staphylococcal sepsis

Variable	*N* = 119
Characteristic	
Median age in years (IQR)	58.5 (46–67)
Female, no. (%)	39 (32.8)
Preexisting conditions, no. (%)	
Diabetes mellitus	42 (35.3)
Kidney disease	23 (19.3)
Liver disease	12 (10.1)
Heart disease	9 (7.6)
Lung disease	7 (5.9)
Neurological disease	5 (4.2)
Alcoholism	4 (3.4)
Hematological disease	4 (3.4)
Cancer	2 (1.7)
Autoimmune disease	2 (1.7)
Median comorbidity index* (IQR)	1 (0–1)
Intervention	
Source control	
Antistaphylococcal antibiotics on first hospital day, no. (%)	109 (91.9%)
Drainage procedure, no. (%)	50 (42.0%)
Days to drainage procedure, median (IQR)	1 (1–4)
Fluid resuscitation and organ support	
Intravenous fluids on first hospital day	97 (81.5%)
Mechanical ventilation	51 (42.9%)
Gas powered ventilator	34 (28.6%)
Electrical ventilator	25 (21.0%)
Vasopressor or inotropic therapy	33 (27.7%)

IQR = interquartile range.

* Comorbidity index is a 10-point score comprising one point each for lung disease, heart disease, kidney disease, liver disease, neurological disease, hematological disease, autoimmune disease, cancer, diabetes, and alcoholism.

**Table 2:** Univariable analysis of predictors of death in severe staphylococcal sepsis

Variable	OR	(95% CI)	*P* value
Baseline			
Age	1.01	0.98–1.04	0.57
Female	1.63	0.65–4.09	0.30
Preexisting condition			
Diabetes mellitus	1.13	0.45–2.85	0.80
Kidney disease	2.03	0.72–5.70	0.18
Liver disease	2.18	0.60–7.94	0.24
Heart disease	3.60	0.89–14.62	0.07
Lung disease	0.64	0.07–5.63	0.69
Neurological disease	0.99	0.11–9.28	0.99
Alcoholism	–		
Hematological disease	1.33	0.13–13.42	0.81
Cancer	–		
Autoimmune disease	4.09	0.25–67.82	0.33
Bacteremia	2.53	0.87–7.37	0.09
Organ failure at enrollment			
Respiratory failure	4.33	1.67–11.23	0.003
Shock	2.33	0.94–5.77	0.07
Thrombocytopenia	1.04	0.39–2.78	0.94
Acute kidney injury	0.99	0.35–2.77	0.98
Acute hepatic injury	0.90	0.24–3.45	0.88
Coagulopathy	5.44	1.78–16.59	0.003
Altered mental status	2.97	0.77–11.50	0.12
Clinical management during hospitalization			
Delayed antibiotics	0.99	0.20–4.99	0.99
Underwent drainage procedure	0.29	0.11–0.85	0.02
Received any intravenous fluid	3.65	0.45–29.36	0.22
Received mechanical ventilation	7.48	2.56–21.88	2.4 × 10^−4^
Received vasopressors or inotropes	3.52	1.38–8.98	0.008
Biomarkers			
IL-6 pg/mL (log_10_)	3.91	1.92–7.94	1.7 × 10^−4^
IL-8 pg/mL (log_10_)	5.86	2.52–13.63	4.1 × 10^−5^

CI = confidence interval; IL = interleukin; OR = odds ratio.

**Table 3:** Multivariable analysis of predictors of death in severe staphylococcal sepsis

Model	Variable	OR	95% CI	*P* value
Clinical variables without IL-6 or IL-8	Sex	1.75	0.62–4.96	0.29
	Respiratory failure	3.94	1.44–10.78	0.007
	Coagulopathy	6.04	1.77–20.57	0.004
	Model pseudo *R*^2^ = 0.15; AIC = 109.1; BIC = 120.2			
With IL-6	Sex	1.87	0.63–5.53	0.26
	Respiratory failure	2.61	0.88–7.78	0.08
	Coagulopathy	4.41	1.09–15.76	0.04
	IL-6 pg/mL (log_10_)	2.74	1.31–5.75	0.008
	Model pseudo *R*^2^ = 0.22; AIC = 102.7; BIC = 116.5			
With IL-8	Sex	3.89	1.05–14.37	0.04
	Respiratory failure	3.40	1.10–10.51	0.03
	Coagulopathy	5.62	1.44–21.87	0.01
	IL-8 pg/mL (log_10_)	7.57	2.71–21.20	< 0.001
	Model pseudo *R*^2^ = 0.31; AIC = 92.8; BIC = 106.6			

AIC = Akaike information criterion; BIC = Bayesian information criterion; CI = confidence interval; IL = interleukin; OR = odds ratio.
