# Supplementary material for: Patient Characteristics, Management, and Predictors of Outcome from Severe Community-Onset Staphylococcal Sepsis in Northeast Thailand: A Prospective Multicenter Study
Source: Am J Trop Med Hyg. 2017 May 3;96(5):1042–9. doi: 10.4269/ajtmh.16-0606 (PMC5417193; doi:10.4269/ajtmh.16-0606)
Supplement: Supplementary file 1 [file SD1.pdf]

Supplemental Table 1

Organ failure in patients with severe staphylococcal sepsis

Organ failure*	N (%)
Shock	45 (37.8)
Acute kidney injury	30 (25.2)
Acute hepatic injury	16 (13.5)
Thrombocytopenia	34 (28.6)
Coagulopathy	16 (13.5)
Respiratory failure	46 (38.7)
Altered mental status	10 (8.4)

Thrombocytopenia is defined as platelets ≤ 100,000/μL. Coagulopathy is defined as prothrombin time ≥ 20 seconds. Respiratory failure is defined as requiring invasive mechanical ventilation. Altered mental status is defined as Glasgow Coma Scale ≤ 12.

*Shock is defined as systolic blood pressure < 90 or use of vasoactive agents. Acute kidney injury is defined as creatinine ≥ 2.0 mg/dL in patients without chronic kidney disease. Acute hepatic insufficiency is defined as bilirubin ≥ 2.0 mg/dL in patients without chronic liver disease.
